# Golgi phosphoprotein 3 sensitizes the tumour suppression effect of gefitinib on gliomas

**DOI:** 10.1111/cpr.12636

**Published:** 2019-05-16

**Authors:** Xu Wang, Zhaohao Wang, Yu Zhang, Yan Wang, Hao Zhang, Shao Xie, Peng Xie, Rutong Yu, Xiuping Zhou

**Affiliations:** ^1^ Institute of Nervous System Diseases Xuzhou Medical University Xuzhou Jiangsu China; ^2^ Department of Neurosurgery Affiliated Hospital of Xuzhou Medical University Xuzhou Jiangsu China; ^3^ The Graduate School Xuzhou Medical University Xuzhou Jiangsu China; ^4^ Jiangsu Center for the Collaboration and Innovation of Cancer Biotherapy Cancer Institute, Xuzhou Medical University Xuzhou Jiangsu China; ^5^ Present address: Department of Neurosurgery PKU Care Luzhong Hospital Zibo Shandong China

**Keywords:** EGFR, gefitinib, Golgi phosphoprotein 3, primary glioma cell, proliferation

## Abstract

**Objectives:**

We previously reported that Golgi phosphoprotein 3 (GOLPH3) promotes glioma progression by inhibiting EGFR endocytosis and degradation, leading to EGFR accumulation and PI3K‐AKT pathway over‐activation. In the current study, we examine whether GOLPH3 affects the response of glioma cells to gefitinib, an EGFR selective inhibitor.

**Materials and Methods:**

The expression of GOLPH3 and EGFR in glioma cells was detected by immunofluorescence and immunoblotting. The cell viability or growth in vitro was determined by CCK‐8, EdU incorporation and clonogenic assays. The primary glioma cells were cultured by trypsin and mechanical digestion. The transwell invasion assay was used to examine the primary glioma cell motility. Intracranial glioma model in nude mice were established to explore the sensitivity of gefitinib to GOLPH3 high cancer cells in vivo.

**Results:**

Both the immortalized and primary glioma cells with GOLPH3 over‐expression hold higher EGFR protein levels on the cell membrane and exhibited higher sensitivity to gefitinib. In addition, primary glioma cells with higher GOLPH3 level exhibited stronger proliferation behaviour. Importantly, GOLPH3 enhanced the anti‐tumour effect of gefitinib in vivo. Consistently, after gefitinib treatment, tumours derived from GOLPH3 over‐expression cells exhibited lower Ki67‐positive and higher cleaved caspase‐3–positive cells than control tumours.

**Conclusions:**

Our results demonstrate that GOLPH3 increases the sensitivity of glioma cells to gefitinib. Our study provides foundation for further exploring whether GOLPH3 high gliomas will be more sensitive to anti‐EGFR therapy in clinic and give ideas for developing new possible treatments for individual glioma patients.

## 1 INTRODUCTION

Glioblastoma multiforme (GBM) is the most aggressive malignant glioma in brain.^1^ Affected patients are usually treated with a combined approach of surgery, chemotherapy and radiation therapy, but the median survival time for GBM patients is approximately 12‐14 months.^2^ Therefore, it is important to elucidate the mechanisms of glioma development and find key molecular targets for the development of effective therapies.^3^

Golgi phosphoprotein 3 (GOLPH3) is a highly conserved protein and mainly located at peripheral membrane of Golgi mature surface, secretory vesicles and tubules of Golgi apparatus, the cytosolic pool and cell membrane.^4^, ^5^, ^6^ Highly conserved from yeast to humans, GOLPH3 is essential for Golgi trafficking and structure maintenance,^7^ protein glycosylation 8 and cell survival after DNA damage.^9^ Recent studies have indicated that GOLPH3 plays an important role in modulation of mitochondrial mass, lipid metabolism and cell mitosis.^10^, ^11^, ^12^ In 2009, GOLPH3 has been identified as a “first‐in‐class Golgi oncoprotein,” which is highly expressed in many human solid tumours and promotes the proliferation of cancer cells through enhancing growth‐factor‐induced mTOR signalling.^13^ Thereafter, GOLPH3 is reported to be up‐regulated in many types of human tumours, such as rhabdomyosarcoma, oesophageal cancer, tongue, gastric cancer and renal cell carcinoma, breast and others, and is related to the poor prognosis of tumours.^14^, ^15^, ^16^, ^17^, ^18^, ^19^, ^20^, ^21^

Our laboratory also carried out a systematic study of GOLPH3 and found that GOLPH3 is up‐regulated in gliomas and promotes glioma cell migration and invasion via Rho A, or the mTOR‐YB1 pathway in vitro.^22^, ^23^ In addition, GOLPH3 promotes glioma cell proliferation and is regulated by protein kinase D2.^24^ Recently, we reported that GOLPH3 promotes glioma cell proliferation via inhibiting endocytosis and degradation of EGFR, thereby activating the PI3K‐AKT‐mTOR signalling pathway.^25^ Gefitinib (also known as Iressa), a selective EGFR inhibitor, is the first molecular target drug in the clinic for treatment of non–small‐cell lung cancer by selectively inhibiting the signal transduction pathway of EGFR tyrosine kinase.^26^ Considering GOLPH3 can inhibit the degradation of EGFR and lead to its accumulation and sustained activation, we, therefore, wonder whether GOLPH3 increases the response of glioma cells to gefitinib.

In this study, we found that both immortalized and cultured primary glioma cells with high GOLPH3 exhibited higher sensitivity to gefitinib treatment. Our findings provide foundation for further exploring whether the glioma patients with high GOLPH3 expression are more sensitive to anti‐EGFR therapy in clinic and develop possible treatment modalities for gliomas.

## 2 MATERIALS AND METHODS

### 2.1 Cell lines, antibodies and reagents

Glioma U251 and U87 cells were purchased from Shanghai Cell bank, Type Culture Collection Committee, Chinese Academy of Sciences. Rabbit‐anti‐GFAP (mab360), mouse‐anti‐EGFR (05‐104) and mouse‐anti‐β‐actin (mab1501) antibodies were purchased from Millipore. Rabbit‐anti‐p‐AKT (4060s), mouse‐anti‐AKT (2920s) and rabbit‐anti‐cleaved caspase‐3 (9661s) were purchased from Cell Signaling Technology. Anti‐p‐EGFR (ab32430) and rabbit monoclonal anti‐GOLPH3 (ab98023) antibodies were purchased from Abcam. Rabbit monoclonal anti‐Ki67 antibody (RM9106) was bought from Thermo Scientific. Alexa Fluor secondary antibodies were from Invitrogen. Gefitinib (S1025) was purchased from Selleck Co.

### 2.2 Establishment of GOLPH3 over‐expression glioma cells

To obtain GOLPH3 over‐expression U251 and U87 cells, the GOLPH3 cDNA was inserted into the pWPXLd backbone (with GFP tag) at BamH I and Mlu I sites. The viruses were propagated in 293 T cells by co‐transfecting corresponding plasmids with the helper plasmids. Glioma cells were infected with lentivirus containing pWPXLd‐GOLPH3 (named GOPH3 thereafter) or pWPXLd (named Vector thereafter).

### 2.3 Cell immunofluorescence

The cells were sequentially incubated with anti‐GFAP or anti‐EGFR primary antibody, and fluorescence‐conjugated secondary antibodies followed by DAPI incubation to stain the nucleus. Cells were photographed under Olympus IX‐71 inverted microscopy.

### 2.4 Cell proliferation assay

Cell viability was assessed using the EdU incorporation assay, cell counting kit‐8 (CCK‐8, Beyotime) and clonogenic assay, respectively. For EdU incorporation assay, cell proliferation was measured by 5‐ethynyl‐20‐deoxyuridine (EdU) incorporation assay using an EdU assay kit (Ribobio) according to the manufacturer's instructions.^25^ For CCK‐8 assay, the absorbance value at 450 nm was measured using a microplate reader (Bio‐Rad). For clonogenic assay, 600 cells were seeded in 60‐mm dish, cultured for 12 days, fixed with methanol, and stained with 0.05% crystal violet. After being photographed with a camera, colonies containing more than 50 cells were counted.^27^

### 2.5 Glioma samples and primary cell culture

The human glioma samples were obtained from Affiliated Hospital of Xuzhou Medical University. All the glioma tissues have been collected immediately after surgical resection and histologically diagnosed according to the World Health Organization grading system. Written informed consent was obtained from the patients, and the study was approved by the Ethic Committee of the hospital.

Glioma tissues (about 1‐3 cm^3^) without electrical coagulation, necrosis or haemorrhage were washed for several times with phosphate‐buffered saline (PBS). The tissues were chopped into mince, suspended with DMEM/F12 medium without serum, and centrifuged at 179 g for 3 minutes. The pellet was resuspended with 0.125% trypsin, incubated in the incubator for 5 minutes, and added with DMEM/F12 with 10% FBS to stop the digestion. The digested tissues were dissociated into single cell suspension mechanically, filtered with 50 microns mesh filter, and cultured with DMEM/F12 containing 10% FBS and penicillin/streptomycin.

### 2.6 Western blot

Equal amount of protein lysates was subjected to 10% SDS‐PAGE and transferred to 0.45 µm pore size PVDF membrane (Millipore). After being blocked with 3% BSA, the membrane was probed with primary antibodies at 4°C overnight and secondary antibodies at room temperature for 1 hour. Bound antibodies were detected by the Pierce ECL Plus Western Blotting Substrate (Thermo Fisher Scientific) and exposed to X‐ray films. Band densities were quantified by software ImageJ. The relative amount of proteins was determined by normalizing the densitometry value of interest to that of the loading control.

### 2.7 Transwell invasion assay

Cell invasion assay was performed using a transwell system (Corning) according to the manufacturer's protocol.^23^

### 2.8 Tumour implantation

Intracranial model of glioma in nude mice was performed according to our previous study.^25^, ^27^ All the in vivo experiments were carried out with ethical committee approval and met the standards required by the guidelines of Xuzhou Medical University. Four‐ to 6‐week‐old female Balb/c athymic nude mice were anaesthetized with 3% chloral hydrate and kept at 37°C. A small burr hole, 1 mm diameter, was drilled with 1.4 mm away from the midline at the right side of the cranium. A 5‐μL microsyringe containing 2.5 μL cell suspension (5 × 10^5^ cells in l‐15 medium) was inserted at 2.3 mm depth from the skull surface. The cells were injected, and microsyringe was held for about 10 minutes before withdrawing. There were 12 mice implanted with GFP (Vector) U87 cells and 14 mice implanted with GFP‐GOLPH3 (GOLPH3) U87 cells. All athymic nude mice were kept in specific pathogen‐free conditions^15^ and cared, according to the animal welfare guidelines of Xuzhou Medical University.

### 2.9 Drug treatment and brain slide preparation

Both the Vector and GOLPH3 cell implanted mice were randomly and evenly divided into two groups separately with each group has six mice (GFP group) or seven mice (GOLPH3 group). According to the results of our pilot study, each mouse was treated with gefitinib (6 mg/kg) or their solvent 0.5% CMC‐Na once every other day by intraperitoneal injection beginning at the 18th day after implantation. At the 30th day after implantation (six treatment), all mice were anaesthetized with chloral hydrate, perfused with 0.1 M PBS and followed by 4% paraformaldehyde (PFA). The fresh‐frozen brains were continuously sectioned at a thickness of 12 μm and stored at −80°C.

### 2.10 Tumour volume and mitosis index

The cryosections of maximal tumour diameter of each group were subjected to haematoxylin and eosin (HE) staining and were used to measure the tumour volume. The tumour volume was calculated according to the formula V = 1/2 ab^2^, with ‘a’ representing the longest diameter and ‘b’ representing the shortest diameter. According to the HE staining, the mitotic index (MI) was assessed by evaluating the percentage of metaphase cells per five high‐power fields randomly.

### 2.11 Statistical analysis

The results were representative of experiments repeated at least three times and expressed as the means ± SEM. Statistical comparisons were performed using Student's *t* test with two tails or ANOVA for multiple comparisons. *P* values < 0.05 were considered statistically significant (*P < 0.05, ** *P* < 0.01, ***P < 0.001). All statistical analyses were performed using Office Excel 2007 (Microsoft Corporation) or SPSS software (SPSS version 18.0).

## 3 RESULTS

### 3.1 GOLPH3 enhances the tumour suppression effect of gefitinib on U251 and U87 cells

**Figure 1:**
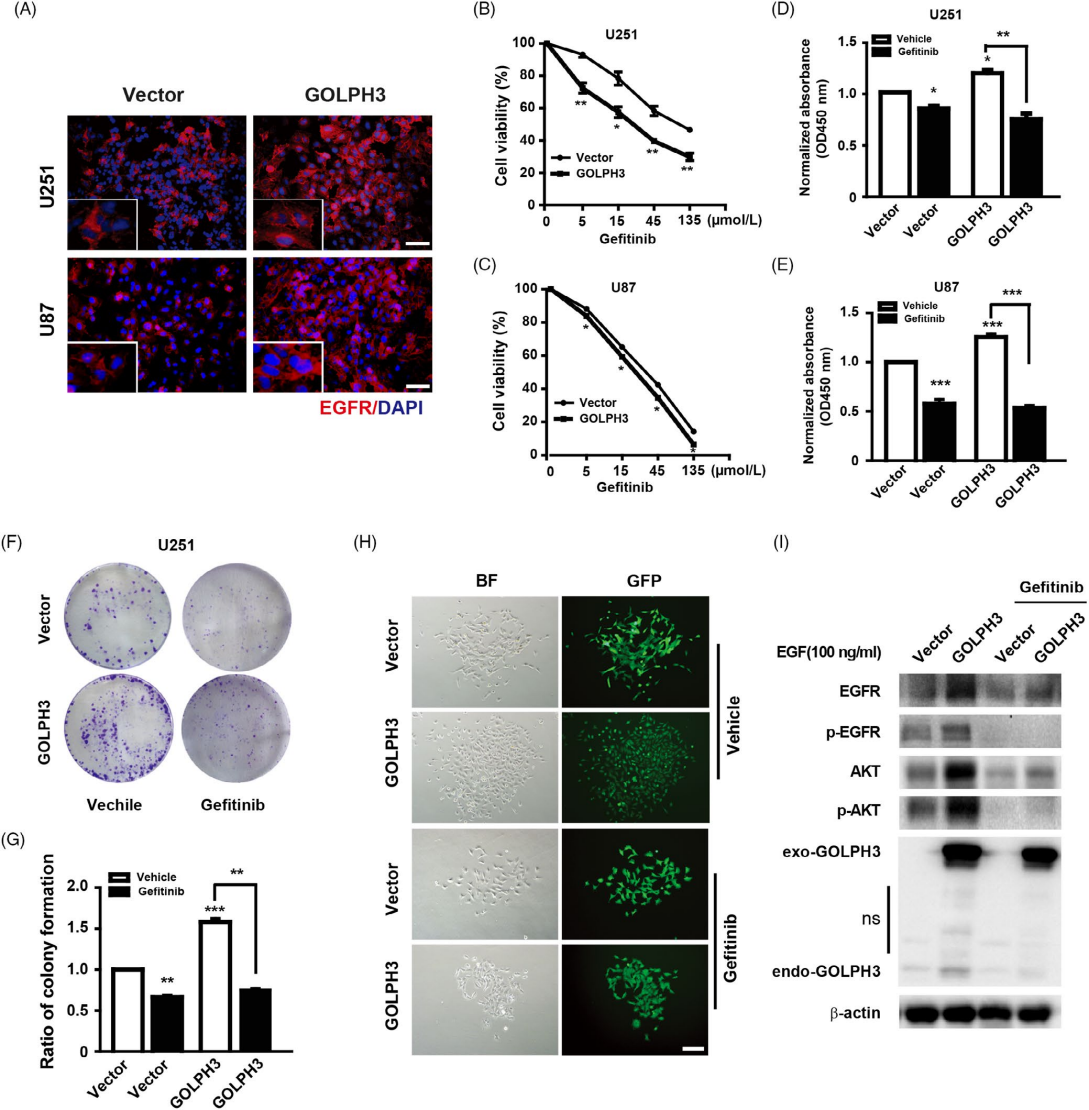
Golgi phosphoprotein 3 (GOLPH3) enhances the tumour suppression effect of gefitinib on U251 and U87 cells. A, Representative images of EGFR expression with or without GOLPH3 over‐expression in U251 and U87 cells. High GOLPH3 expression cells showed higher EGFR protein levels, which mainly located at the cell membrane. Red: EGFR; Blue: DAPI. Scale bar: 100 μm. B&C Examined by CCK 8 assay, GOLPH3 over‐expression sensitized the anti‐proliferation effect of gefitinib on U251 (B) and U87 (C) cells in a dose‐dependent manner. (D&E) GOLPH3 over‐expression cells exhibited higher proliferation inhibition effect of gefitinib (30 μM) on U251 (D) and U87 (E) cells. F, Representative images of clonogenic assay after gefitinib treatment with or without GOLPH3 over‐expression. GOLPH3 over‐expression cells showed stronger colony formation inhibition after gefitinib treatment. G, Quantitative results of the clonogenic assay of U251 cells. H Bright field (BF) and fluorescent (GFP) images of typical single colony formed by U251 cells infected with the indicated GFP‐tagged lentivirus. Scale bar: 200 μm. I, Representative immunoblots of the U251 cell extracts of the vector control and GOLPH3 over‐expression cells with or without gefitinib (30 µM) treatment probed with indicated antibodies. ns: non‐specific. *P < 0.05, **P < 0.01, ***P < 0.001

We previously reported that GOLPH3 inhibits the endocytosis of EGFR and enhances the total protein level of EGFR.^25^ Here, we firstly checked the protein level of EGFR on the plasma membrane using immunofluorescence in the GOLPH3 over‐expression glioma cells (Figure S1). As shown in Figure 1A, the U251 and U87 glioma cells with GOLPH3 over‐expression exhibited higher EGFR level on the plasma membrane. Thereafter, the proliferation of GOLPH3 over‐expression U251 and U87 glioma cells, with or without gefitinib treatment, was detected by CCK8 and colony formation assay, respectively. Firstly, we found that both the cell viability of the vector and the GOLPH3 over‐expression glioma cells decreased in a dose‐dependent manner after gefitinib treatment (Figure 1B, 1). Excitingly, the GOLPH3 over‐expression U251 cells exhibited higher sensitivity to gefitinib and the IC50 was about 35.25 μM, which was significantly lower than that of the vector group (105.1 μM). Consistently, the IC50 of gefitinib in GOLPH3 over‐expression U87 cells was about 24.21 μM, which was significantly lower than that of the vector group (35.88 μM). In addition, after gefitinib (30 μM) treatment, both the proliferation of the vector and the GOLPH3 over‐expression cells decreased (Figure 1D, 1). Interestingly, after gefitinib treatment, the cell proliferation of GOLPH3 high U251 cells decreased by 37.65%, which was more significant than that of vector cells (only 15.73% decrease, Figure 1D). Similarly, after gefitinib treatment, the cell proliferation of GOLPH3 high U87 cells decreased by 56.8%, which was more striking than that of vector cells (40% decrease, Figure 1E).

Furthermore, the above results were repeated using the colony formation assay (Figure 1F, G). The relative colony formation rate difference between the vector group was 33.7% after gefitinib treatment, while that between GOLPH3 over‐expression group was 52.18%. Moreover, typical single colony formed by U251 cells infected with the indicated GFP‐tagged lentivirus showed that, after gefitinib treatment, the clone size difference between GOLPH3 high cells was more impressive than that of the vector group (Figure 1H). Therefore, our findings indicate that GOLPH3 enhances the tumour suppression effect of gefitinib on U251 and U87 cells. Importantly, we detected the EGFR level and its downstream signalling (AKT activity as an index) in the vector control and GOLPH3 over‐expression cells before and after gefitinib treatment. As shown in Figure 1I and Figure S2, the total level of EGFR and its downstream activity obviously increased after GOLPH3 over‐expression without gefitinib treatment, consistent with our previous report.^25^ In addition, the activity of EGFR and AKT dramatically decreased after gefitinib treatment in both group. Furthermore, compared to the vector group, the activity of EGFR and AKT decreased more strikingly in GOLPH3 over‐expression cells after gefitinib treatment, indicating the clear relationship between EGFR level induced by GOLPH3 and the gefitinib sensitivity and in line with the result of cell proliferation. The above result indicates that the GOLPH3 acted through PI3K‐AKT pathway and GOLPH3 high cells were more sensitive to gefitinib treatment indeed.

### 3.2 GOLPH3 over‐expression promotes the proliferation and invasion of primary glioma cells

**Figure 2:**
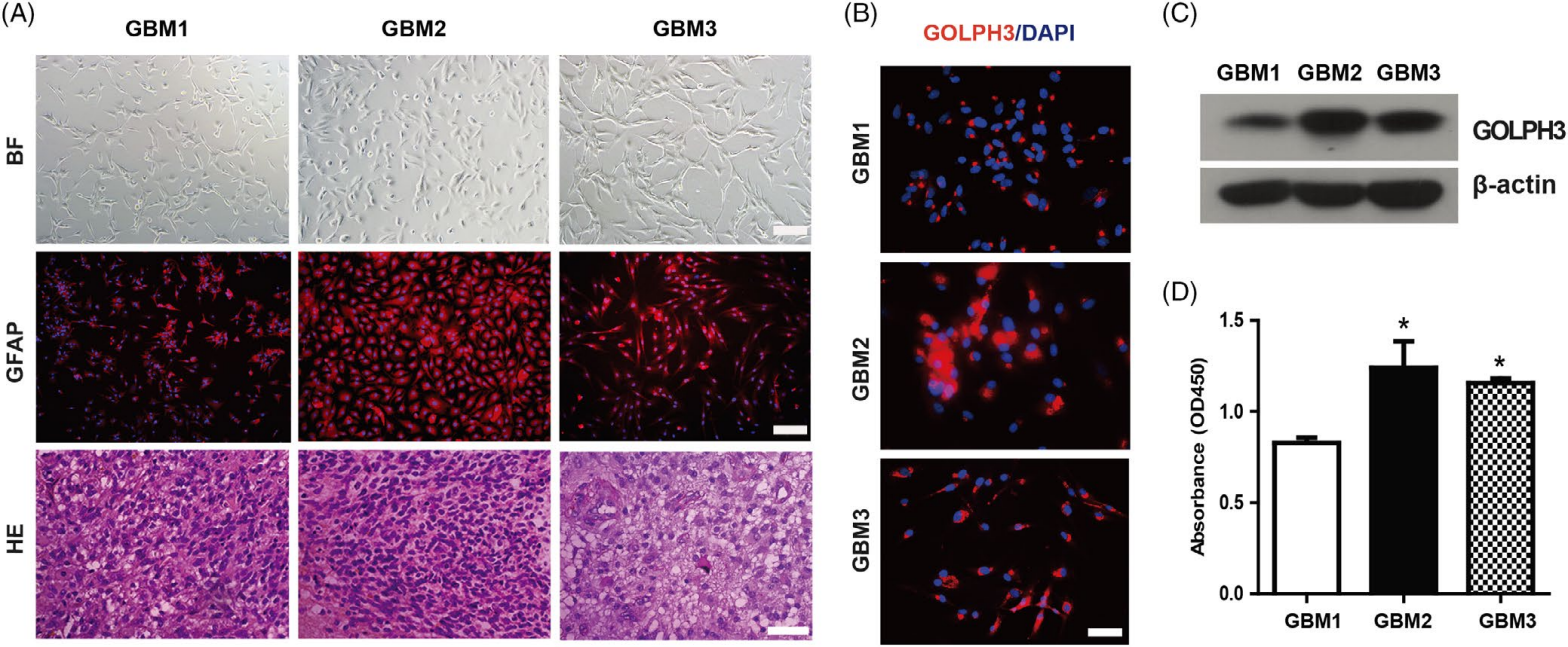
Primary glioma cells with high Golgi phosphoprotein 3 (GOLPH3) level grow faster. A, Representative images of the cultured primary glioma cells (named GBM1, GBM2, GBM3) and corresponding GFAP staining at passage 1 (P1). Primary glioma cells derived from different patients showed diverse cell morphology, such as stellate, spindle and polygonal. BF: bright field. Red: GFAP. Blue: DAPI. Scale bar: 200 μm. HE staining for the corresponding glioma tissue sections. Scale bar: 50 μm. B, Representative immunofluorescence images of GOLPH3 staining in three strains of primary glioma cells at P1. GOLPH3 was mainly located at the perinuclear region. Red: GOLPH3. Blue: DAPI. Scale bar: 100 μm. C, The protein level of GOLPH3 in three primary glioma cells was examined by Western blot assay. D, The GBM2 and GBM3 cells showing higher GOLPH3 levels grew faster. *P < 0.05

Compared with the immortalized cells, cultured primary glioma cells retain the original genetic characteristics of the tumour in nature, reflect the in vivo growth features and are an ideal model for gene expression and drug toxicity in vitro.^28^, ^29^, ^30^ To address whether the above phenomena observed in immortalized glioma cells could reflect the characteristics of gliomas in vivo, we cultured the primary glioma cells using freshly resected glioma samples. Using the enzyme digestion method, we successfully cultured 18 cases and got eight cases of longer subculture. We selected three strains of primary glioblastoma cells (named GBM1, GBM2 and GBM3, respectively) with good condition to perform the following experiments. As shown in Figure 2A, the nucleus division was obvious in the corresponding HE staining of GBM1, GBM2 and GBM3 tumour tissue. In addition, the primary cells expressed good glial acidic protein (GFAP), the hallmark of gliomas (Figure 2A). Furthermore, the primary cells expressed GOLPH3, which was rich in the perinuclear region (Figure 2B, 2). Excitingly, the GBM2 and GBM3 cells with higher GOLPH3 levels grew faster than GBM1, which has lower GOLPH3 level (Figure 2D), in line with our previous study.^25^

**Figure 3:**
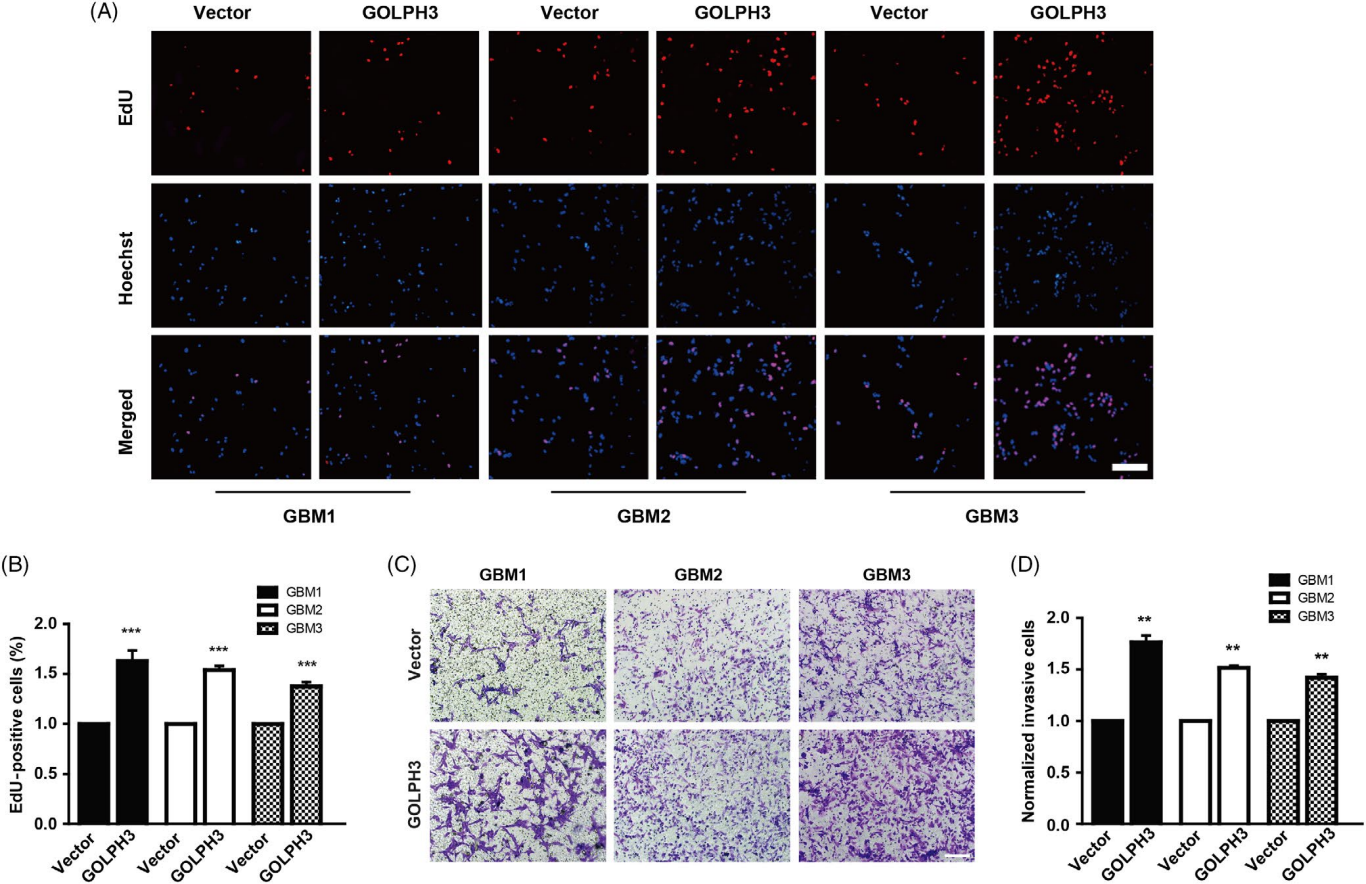
Golgi phosphoprotein 3 (GOLPH3) promotes the proliferation and invasion of primary glioma cells. A, Representative images of EdU incorporation assay in three strains of primary glioma cells over‐expressing GOLPH3 at P5. Scale bar: 200 μm. B, Quantitative analysis of EdU positive cells. C, Representative images of transwell invasion assay in three strains of primary glioma cells over‐expressed with GOLPH3 at P5. Scale bar: 200 μm. (D) Quantitative analysis of invasive cells. **P < 0.01, ***P < 0.001

Next, after over‐expressing GOLPH3 in the primary cells (Figure S3), GOLPH3 over‐expression GBM cells exhibited higher proliferation ability, which showed higher percentage of EdU positive cells, than that of the vector cells (Figure 3A, 3). Furthermore, examined by matrigel‐precoated transwell assay, the number of invasive cells increased markedly after GOLPH3 over‐expression (Figure 3C, 3). These results demonstrate that GOLPH3 over‐expression promotes the proliferation and invasion of primary glioma cells.

### 3.3 GOLPH3 enhances the tumour suppression effect of gefitinib on primary glioma cells

**Figure 4:**
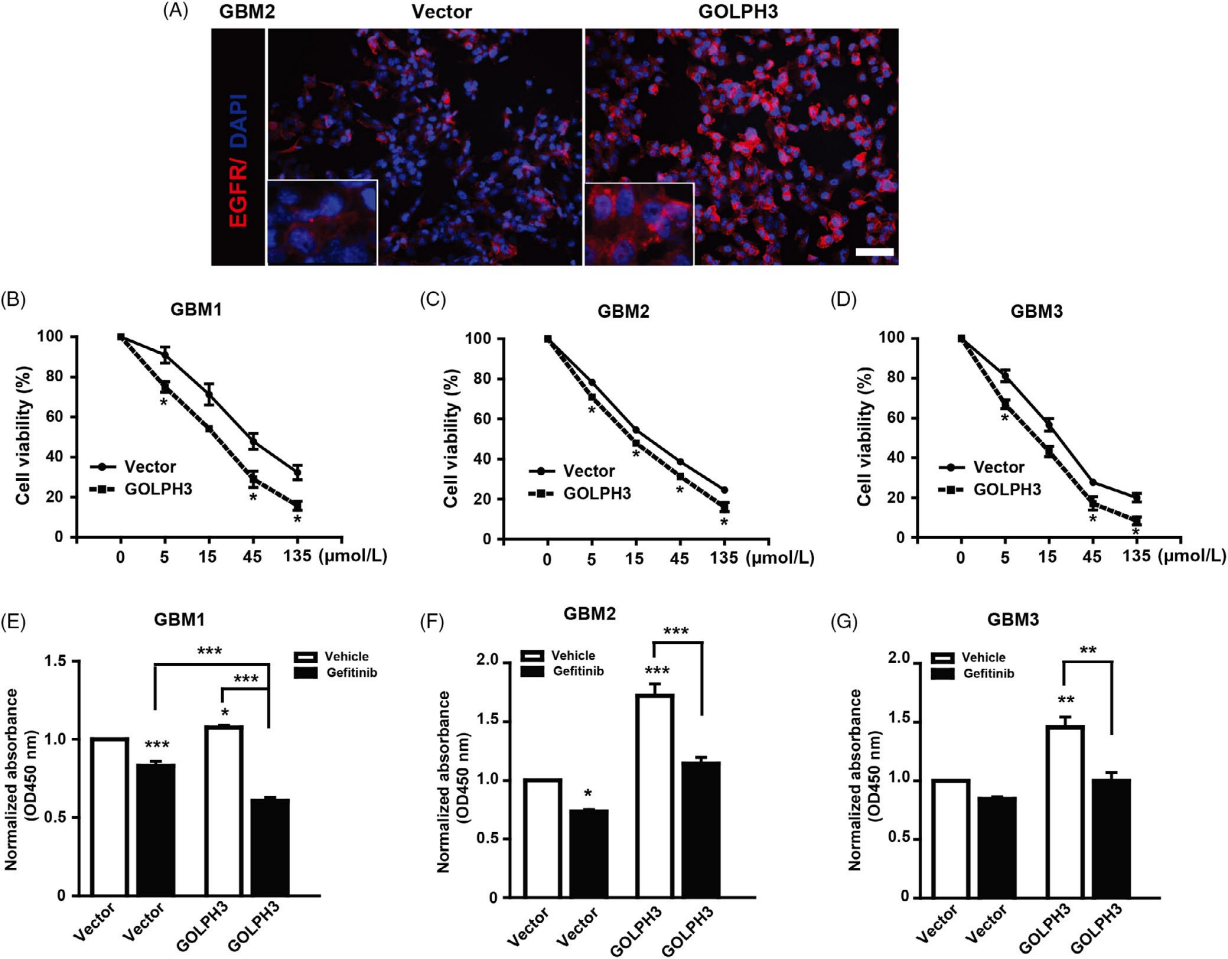
Golgi phosphoprotein 3 (GOLPH3) sensitizes the anti‐tumour effect of gefitinib on primary glioma cells. A, Representative images of EGFR expression with or without GOLPH3 over‐expression in GBM2 cells at P5. High GOLPH3 expression cells showed higher EGFR protein levels, which mainly located at the cell membrane. Red: EGFR; Blue: DAPI. Scale bar: 100 μm. B‐D, Examined by CCK 8 assay, GOLPH3 over‐expression sensitized the anti‐proliferation effect of gefitinib on three strains of primary glioma cells in a dose‐dependent manner. E‐G, GOLPH3 over‐expression cells exhibited higher proliferation inhibition effect of gefitinib (30 μM) on three strains of primary glioma cells than that of control cells (Vector). *P < 0.05, **P < 0.01, ***P < 0.001

Similar to the results found in U251 cells, the protein level/intensity of EGFR increased in GOLPH3 over‐expression GBM2 primary glioma cells and mainly located at cell membrane (Figure 4A). As shown in Figure 4B‐D, after gefitinib treatment, the cell viability of the vector and the GOLPH3 over‐expression primary GBM cells decreased in a dose‐dependent manner. Excitingly, the GOLPH3 over‐expression GBM1 cells exhibited higher sensitivity to gefitinib and the IC50 was about 18.56 μM, which was lower than that of the vector group (54.35 μM). Consistently, the IC50 of gefitinib in GOLPH3 over‐expression GBM2 and GBM3 cells was about 14.49 μM and 12.69 μM, which was significantly lower than that of the corresponding vector group (27.98 μM and 21.82 μM).

We further found that gefitinib (30 μM) treatment decreased the proliferation of both the vector and the GOLPH3 over‐expression primary cells (Figure 4E‐G). In addition, after gefitinib treatment, the cell proliferation of GOLPH3 high GBM1 cells decreased by 43.52%, which was more striking than that of vector cells (only 17% decrease, Figure 4E). Similarly, after gefitinib treatment, the cell proliferation of GOLPH3 high GBM2 cells decreased by 33.72%, which was more striking than that of vector cells (27% decrease, Figure 4F). After gefitinib treatment, the inhibition rate difference between the vector GBM3 cells was 16%, while that of the GOLPH3 over‐expression group was 31% (Figure 4G). Therefore, our findings indicate that GOLPH3 enhances the tumour suppression effect of gefitinib on primary glioma cells.

### 3.4 GOLPH3 sensitizes the anti‐tumour effect of gefitinib in vivo

**Figure 5:**
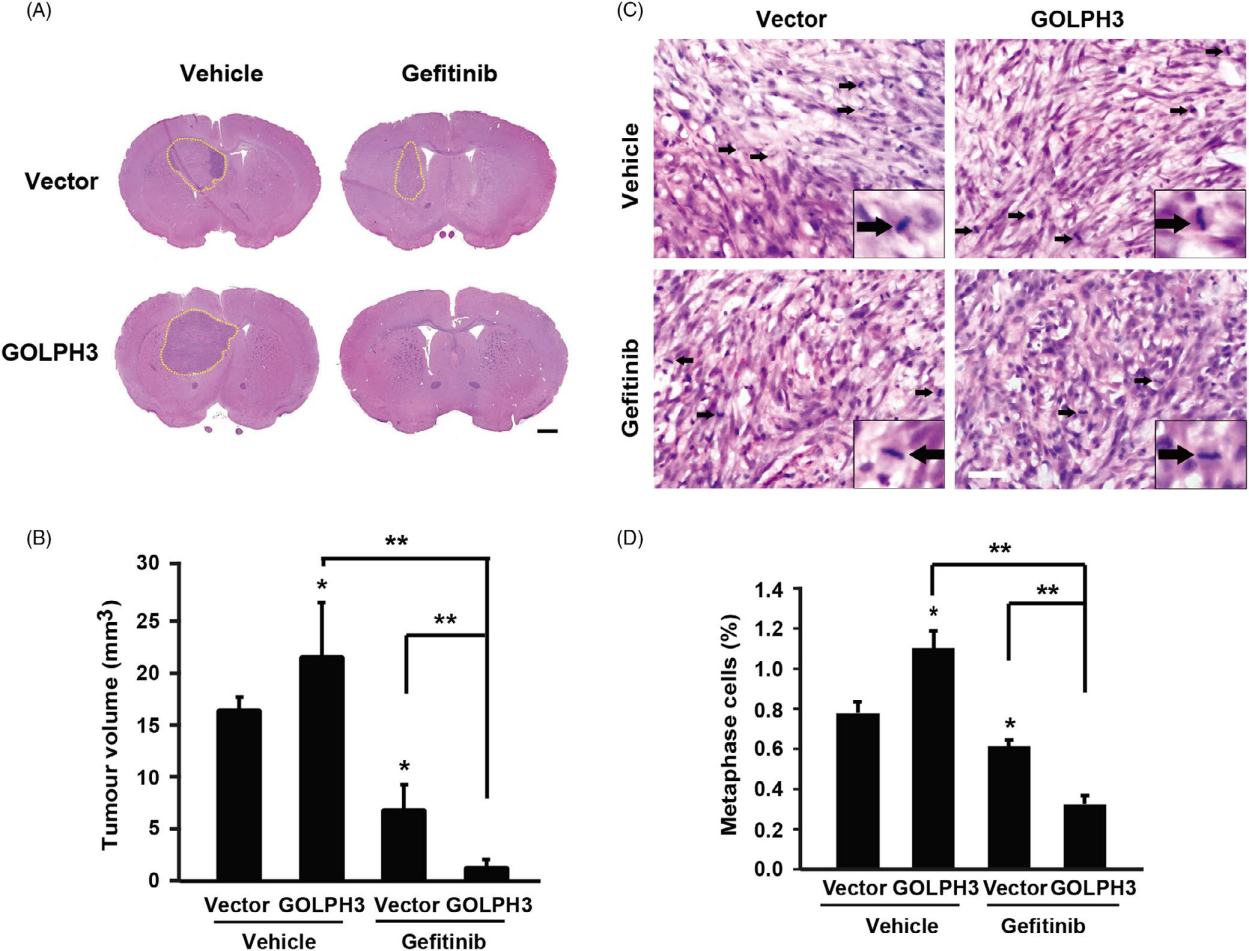
Golgi phosphoprotein 3 (GOLPH3) enhances the tumour suppression effect of gefitinib in vivo. A, Representative images of HE staining of tumours derived from Vector and GOLPH3 over‐expression U87 cells with or without gefitinib treatment. Scale bar: 1 mm. B, Quantitative analysis of the tumour volume. Vector + Vehicle = 15.31 ± 1.38 mm^3^; GOLPH3 + Vehicle = 20.25 ± 5.12 mm^3^; Vector + gefitinib = 6.15 ± 2.47 mm^3^; GOLPH3 + gefitinib = 0.85 ± 0.85 mm^3^. C, The Mitotic Index was determined by counting the number of metaphase cells per five high‐power fields. Scale bar: 25 μm. D, Quantitative analysis of C. Vector + Vehicle = 0.79 ± 0.06%; GOLPH3 + Vehicle = 1.11 ± 0.09%; Vector + gefitinib = 0.62 ± 0.03%; GOLPH3 + gefitinib = 0.33 ± 0.04%. *P < 0.05; **P < 0.01

Next, we transplanted the U87 cells into the right striatum of nude mice by stereotactic technique to establish intracranial glioma model. As shown in Figure 5A, the HE staining showed visible tumour formation in right striatum with higher cell density than normal brain tissues. Meanwhile, the tumour derived from GOLPH3 over‐expression cells was larger than that derived from vector cells (Figure 5A, 5), indicating that GOLPH3 promoted tumour growth. In addition, gefitinib treatment caused a significant decrease of tumour volume both in the vector and GOLPH3 over‐expression groups (Figure 5A, 5). Interestingly, GOLPH3 over‐expression group exhibited higher sensitivity to gefitinib treatment, elucidated by the most striking tumour volume decrease (Figure 5B). Furthermore, as shown in Table 1, after gefitinib treatment, there was only one mouse in seven bearing tumour in GOLPH3 over‐expression group, while every mouse bore tumour in the control group. The tumour formation rate significantly decreased in GOLPH3 over‐expression group, implying that over‐expression of GOLPH3 enhanced the anti‐tumour effect of gefitinib in vivo.

**Table 1:** Summarizing data from the gefitinib‐treatment xenograft studies at day 30 after 6 doses

Treatment	Fold change	%TGI	TBR
Vector			
Vehicle	1	0	6/6
Gefitinib	0.38	62.13	5/6
GOLPH3			
Vehicle	1	0	7/7
Gefitinib	0.04	95.65	1/7

Abbreviations: %TGI, the percentage of tumour growth inhibition; TBR, tumour burden rate.

The classic characteristic of the transplanted tumour, such as nuclear atypia and mitosis (Figure 5C), was seen in the HE staining. By counting the metaphase cells (mitotic index), we found that the percentage of metaphase cells of tumours derived from GOLPH3 over‐expression cells was 40.5% higher than that of vector group (Figure 5C, 5). Interestingly, after gefitinib treatment, the percentage of metaphase cells of tumours derived from GOLPH3 over‐expression group decreased by 71.17%, which was more striking than that of the control group (21.52% decrease, Figure 5D), indicating that over‐expression of GOLPH3 enhanced the anti‐tumour effect of gefitinib in vivo.

### 3.5 GOLPH3 over‐expression strengthens the proliferation inhibition and apoptosis promotion effect of gefitinib in vivo

**Figure 6:**
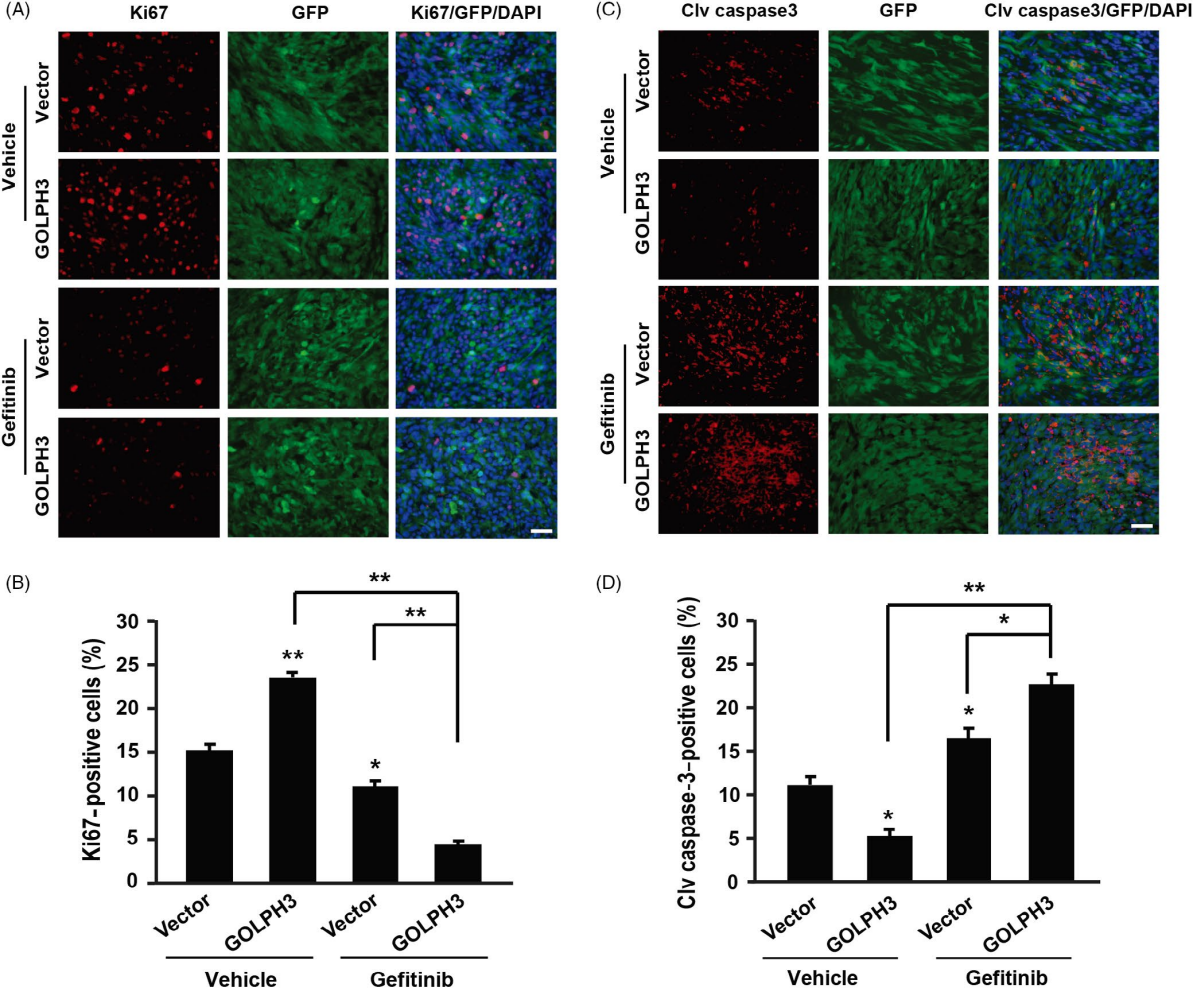
Golgi phosphoprotein 3 (GOLPH3) over‐expression enhances cell proliferation inhibition and apoptosis promotion induced by gefitinib treatment in vivo. A, Representative images of Ki67 staining of tumours derived from Vector and GOLPH3 over‐expression cells with or without gefitinib treatment. Scale bar: 25 µm. B, Quantitative analysis of the Ki67‐positive cells. The percentage of Ki67‐positive cells of each groups were as follows: Vector + Vehicle = 15.35 ± 0.69%; GOLPH3 + Vehicle = 23.81 ± 0.53%; Vector + gefitinib = 11.19 ± 0.70%; and GOLPH3 + gefitinib = 4.45 ± 0.43%. C, Representative images of cleaved (Clv) caspase‐3 staining of tumours derived from Vector and GOLPH3 over‐expression cells with or without gefitinib treatment. Scale bar: 25 µm. D, Quantitative analysis of the Clv caspase‐3–positive cells. The percentage of Clv caspase‐3–positive cells of each groups were as follows: Vector + Vehicle = 11.28 ± 1.0%; GOLPH3 + Vehicle = 5.35 ± 0.76%; Vector + gefitinib = 16.74 ± 1.15%; and GOLPH3 + gefitinib = 23.00 ± 1.26%. *P < 0.05, **P < 0.01

Next, we found that the percentage of Ki67‐positive cells in the tumours derived from GOLPH3 over‐expression cells was 54.54% higher than that of vector group (Figure 6A, 6). In addition, gefitinib treatment caused significant decrease in the percentage of Ki67‐positive cells both in the vector and GOLPH3 over‐expression groups (Figure 6A, 6). Interestingly, after gefitinib treatment, the percentage of Ki67‐positive cells of GOLPH3 over‐expression group decreased by 81.31%, which was more significant than that of the control group (27.1% decrease, Figure 6B), indicating that GOLPH3 high cells exhibited higher sensitivity to gefitinib.

Cleaved caspase‐3 is a most frequently used marker for cell apoptosis. In our system, we found that the percentage of cleaved caspase‐3–positive cells in the tumours derived from GOLPH3 over‐expression group was lower than that derived from vector cells (Figure 6C, 6), indicating that GOLPH3 inhibited tumour cell apoptosis. Gefitinib treatment caused significant increase in the percentage of cleaved caspase‐3–positive cells both in the vector and GOLPH3 over‐expression groups (Figure 6C, 6). Interestingly, the percentage of cleaved caspase‐3–positive cells of GOLPH3 over‐expression group increased by 329.91%, which was more striking than that of the control group (48.4% increase, Figure 6D).

## 4 DISCUSSION

In 2009, Scott and his colleagues reported that GOLPH3 activates the mTOR signalling and therefore enhances the sensitivity of cancer cells to rapamycin.^13^ We previously found that GOLPH3 inhibits the endocytosis and degradation of EGFR, leading to the accumulation and continuing activation of EGFR and the consequent proliferation of gliomas.^25^ In addition, we also found that GOLPH3 promotes the retrograde trafficking of Wls and thereafter Wnt2b secretion to enhance glioma cell growth.^31^ In this study, we found that GOLPH3 over‐expression glioma cells exhibited higher sensitivity to gefitinib treatment in vitro and in vivo. Examined by EGFR immunofluorescence, we found that, either in the U251 or in the primary cultured glioma cells, the EGFR protein levels increased and accumulated at the cell membrane after GOLPH3 over‐expression. In addition, after gefitinib treatment, the level of p‐EGFR and p‐AKT in GOLPH3 over‐expression cells decreased dramatically than that of the vector cells, indicating that inhibition of EGFR in GOLPH3 high cells by gefitinib could better block the downstream PI3K‐AKT signalling, leading to stronger cell growth inhibition. Furthermore, our results also indicate that the promotion effect of GOLPH3 on glioma proliferation is mainly through enhancing the function of EGFR related pathways.

Notably, the level of AKT dramatically increased without gefitinib treatment in GOLPH3 over‐expression cells (Figure 1I and Figure S2). Because GOLPH3 was involved in vesicle trafficking,^10^, ^32^ which is important for the mature and function of proteins, we deduce that, without gefitinib treatment, high GOLPH3 level and activity lead to high protein trafficking and caused the increase of AKT. Interestingly, the total level of EGFR and AKT decreased after gefitinib treatment in GOLPH3 over‐expression group. However, we have not known the exact mechanism which caused this result so far as the molecular mechanism of gefitinib is to selectively bind to the adenosine triphosphate (ATP)‐binding site of the EGFR tyrosine kinase domain which does not influence total EGFR level. We guess that, in the presence of gefitinib, AKT decrease in the GOLPH3 over‐expression group may be caused by the abnormal accumulated and un‐activated AKT in the inner cell membrane triggering the feedback degradation.^33^ According to the literature,^34^, ^35^ EGFR can be endocytosed through clathrin‐dependent (at low doses of EGF stimulation) and clathrin‐independent or lipid raft‐dependent (at high doses of EGF stimulation) pathways. At high concentrations of EGF (20 ng/mL), a substantial fraction of the receptor is endocytosed through a lipid raft (caveolae)‐dependent route as the receptor becomes ubiquitinated. Because GOLPH3 was important to lipid metabolism 10, 32 and our results were from 100 ng/mL EGF stimulation situation, we deduce that, in the presence of gefitinib, high GOLPH3 triggering the lipid raft‐dependent endocytosis and degradation of the accumulated and un‐activated EGFR on the cell membrane with an unknown mechanism. Our above interesting findings deserve further experiments to explore the mechanism in the future. According to our results that both the level of p‐EGFR and total EGFR decreased after gefitinib treatment in GOLPH3 over‐expression cells, we guess that the higher anti‐proliferative effect by gefitinib on GOLPH3 over‐expression cells may be caused by gefitinib‐induced EGFR activity inhibition and/or by gefitinib‐induced EGFR decrease.

Notably, we surprisingly found that, after gefitinib treatment, the tumour derived from GOLPH3 over‐expression cells significantly shrank and almost disappeared. In our system, after gefitinib treatment, there was only one in seven mice exhibited tumour in GOLPH3 over‐expression group, while every mouse bore tumour in the control group. The above result indicated that the sensitization effect of GOLPH3 over‐expression on gefitinib treatment in in vivo system was stronger than that found in in vitro system. In addition to the EGFR level, the total number of tumour cells and other microenvironment factors in vivo will affect the effect of gefitinib, which may cause the sensitization effect in vivo higher than that displayed in vitro. Our findings further support the importance of microenvironment in tumour progression.^36^, ^37^

Primary glioma cells retain the original genetic and growth characteristics of the tumour in the body and are the ideal testing model for gene expression and drug toxicity in vitro. We successfully cultured and identified several strains of primary glioma cells, which expressed higher GFAP, the molecular marker of gliomas, and higher GOLPH3 in the perinuclear region. By using the cultured primary glioma cells, we found that GOLPH3 promoted primary glioma cell growth and migration. Furthermore, GOLPH3 also increased the sensitivity of primary glioma cell to gefitinib. Theoretically, using the primary glioma cells to perform the above experiment in vivo will have more practical significance. However, as the primary glioma cells are ageing fast and we cannot collect enough cells to do transplantation, we are temporarily unable to complete this experiment in primary glioma cells in vivo. But we believe that, with the time and technology optimization, the results of this study will be presented in the near future.

Theoretically, our study found that GOLPH3 high cells exhibited higher sensitivity to gefitinib treatment, indicating the great potential that the GOLPH3 high GBM patients may benefit from anti‐EGFR therapy. However, maybe because the wild‐type EGFR possess lower affinity for gefitinib than that of the Exon19 del/L858R mutant,^38^, ^39^ the IC50 of gefitinib in glioma cells both in our study and others^40^ was higher than those in other type of cancers such as NSCLC with EGFR mutation.^38^ Therefore, it is hard to directly examine the clinical response of gefitinib in GBM patients with high GOLPH3 now. Fortunately, there are some other anti‐EGFR therapy such as cetuximab, nimotuzumab and panitumumab targeting the extracellular domain of EGFR on the way. Therefore, it will be interest to test whether GOLPH3 high patients has better response to these agents and benefit the GOLPH3 high GBM patients in the future.

Taken together, our results demonstrate that GOLPH3 enhances the anti‐tumour effect of gefitinib to glioma cells. It will be very interesting to further explore whether GOLPH3 high glioma patients will be more sensitive to anti‐EGFR therapy and provide ideas for developing new possible treatments for individual glioma patients.

## CONFLICTS OF INTEREST

The authors declare no conflict of interest.

## Data Availability

The data that support the findings of this study are available from the corresponding author upon reasonable request.
