# Supplementary material for: Golgi phosphoprotein 3 sensitizes the tumour suppression effect of gefitinib on gliomas
Source: Cell Prolif. 2019 May 16;52(4):e12636. doi: 10.1111/cpr.12636 (PMC6669003; doi:10.1111/cpr.12636)
Supplement: Supplementary file 1 [file CPR-52-e12636-s001.docx]

**Supporting information**

**
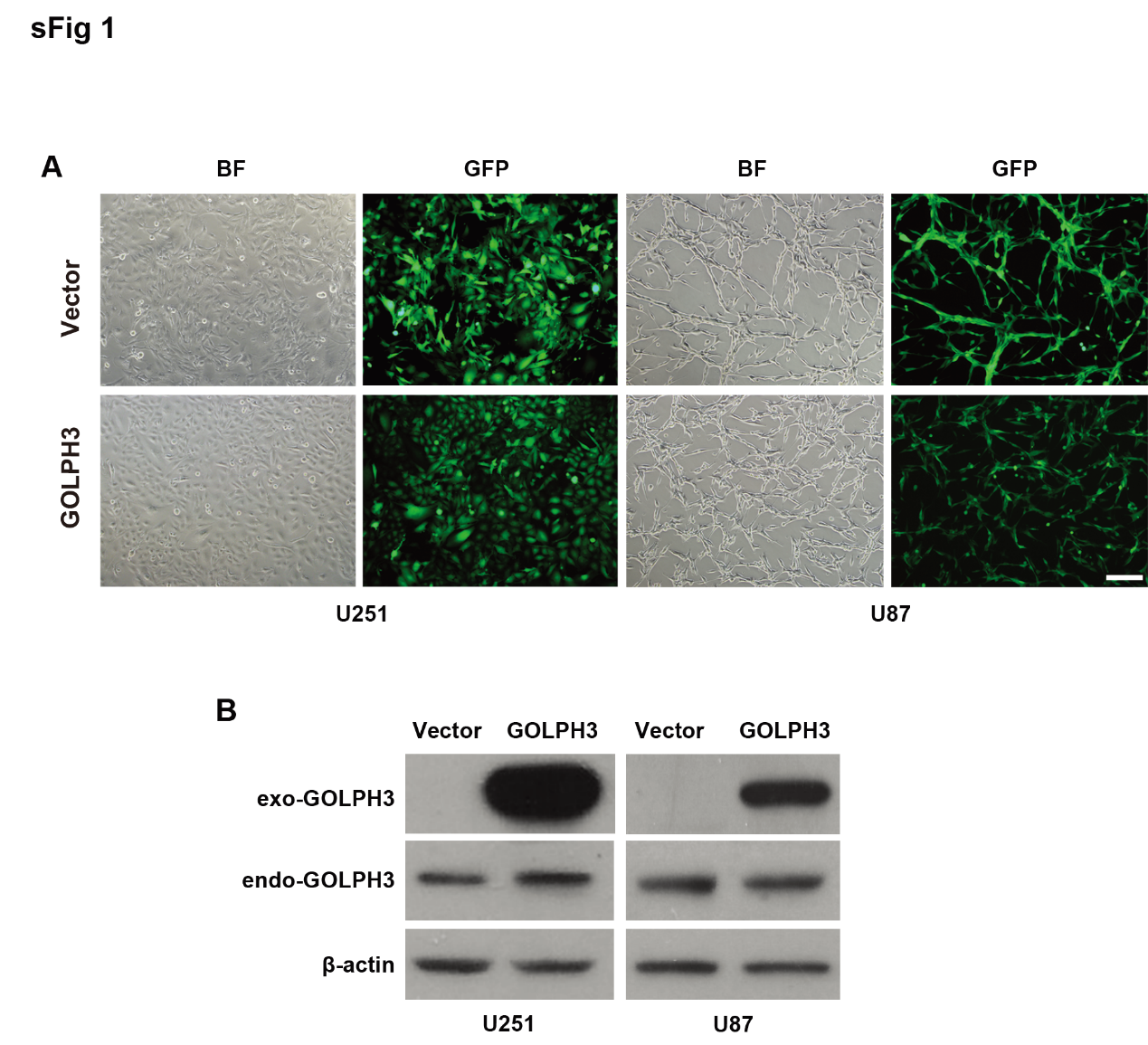
**

**sFig 1. Construction of GOLPH3 over-expression U251 and U87 glioma cells**

**A** The bright field (BF) and fluorescent (GFP) images showed the GOLPH3 over-expression efficiency in U251 and U87 cells. The fluorescence efficiency of cells was more than 95%. Scale bar: 200μm. **B** The GOLPH3 over-expression efficiency in U251 and U87 cells was detected by Western blot assay.

**sFig 2.** **(A &B)** Immunoblots of the U251 cell extracts of the vector control and GOLPH3 over-expression cells with or without gefitinib (30µM) treatment probed with indicated antibodies. ns: non-specific.

**
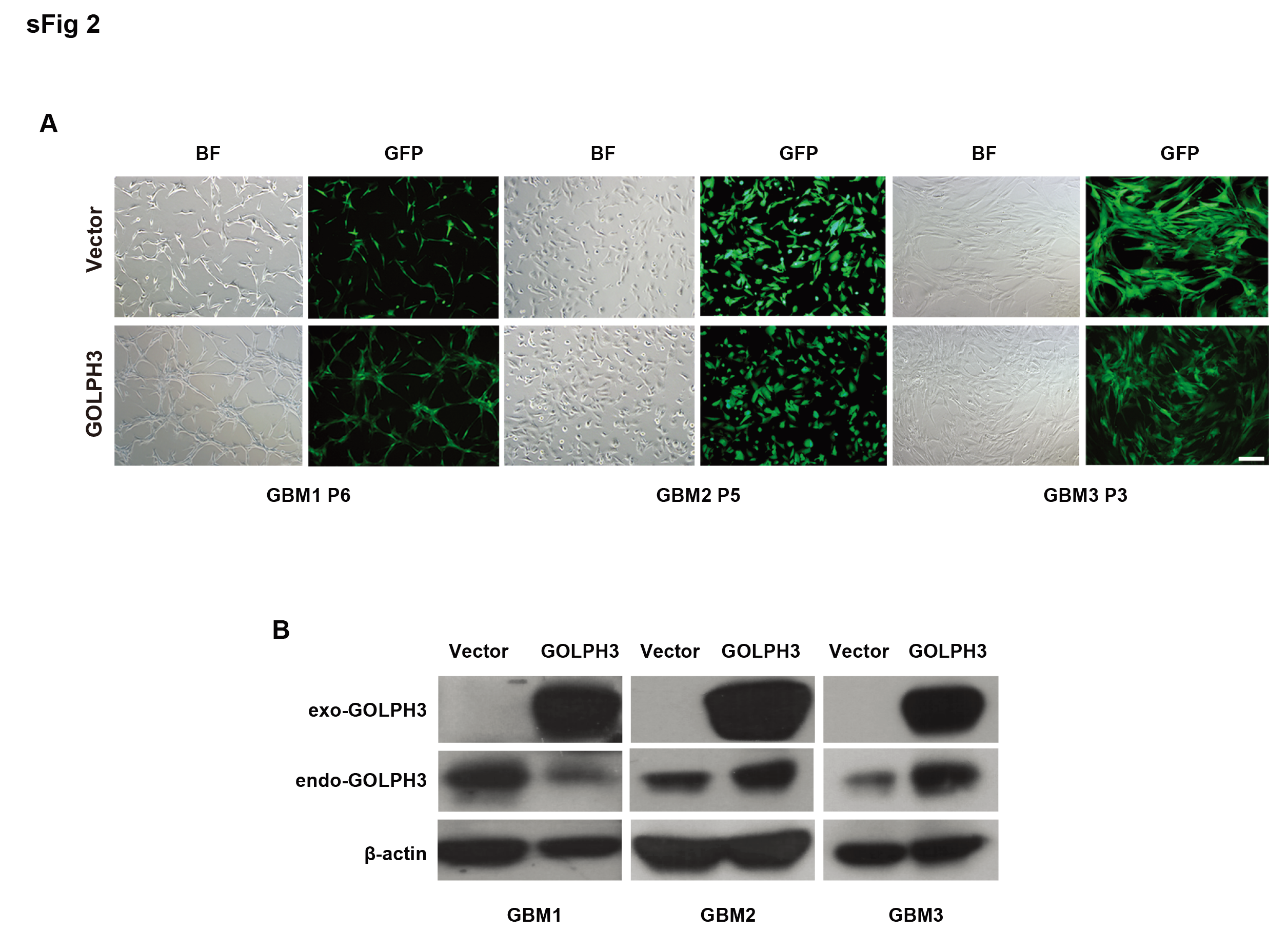
**

**sFig 3. Construction of GOLPH3 over-expression primary glioma cells**

**A** The bright field (BF) and fluorescent (GFP) images showed the GOLPH3 over-expression efficiency in three strains of primary glioma cells at different passage (GBM1 at P6, GBM2 at P5, GBM3 at P3). The fluorescence efficiency of cells was more than 95%. Scale bar: 200μm. **B** The GOLPH3 over-expression efficiency in primary glioma cells was detected by Western blot assay.
